# Development and Assessment of Plant-Based Synthetic Odor Baits for Surveillance and Control of Malaria Vectors

**DOI:** 10.1371/journal.pone.0089818

**Published:** 2014-02-24

**Authors:** Vincent O. Nyasembe, David P. Tchouassi, Hillary K. Kirwa, Woodbridge A. Foster, Peter E. A. Teal, Christian Borgemeister, Baldwyn Torto

**Affiliations:** 1 Behavioral and Chemical Ecology Department, International Centre of Insect Physiology and Ecology (*icipe*), Nairobi, Kenya; 2 Department of Ecology and Organismal Biology, The Ohio State University, Columbus, Ohio, United States of America; 3 Center for Medical, Agricultural, and Veterinary Entomology, U.S. Department of Agriculture, Agricultural Research Service, Gainesville, Florida, United States of America; 4 Center for Development Research, University of Bonn, Bonn, Germany; University of California Davis, United States of America

## Abstract

**Background:**

Recent malaria vector control measures have considerably reduced indoor biting mosquito populations. However, reducing the outdoor biting populations remains a challenge because of the unavailability of appropriate lures to achieve this. This study sought to test the efficacy of plant-based synthetic odor baits in trapping outdoor populations of malaria vectors.

**Methodology and Principal Finding:**

Three plant-based lures ((*E*)-linalool oxide [LO], (*E*)-linalool oxide and (*E*)-β-ocimene [LO + OC], and a six-component blend comprising (*E*)-linalool oxide, (*E*)-β-ocimene, hexanal, β-pinene, limonene, and (*E*)-β-farnesene [Blend C]), were tested alongside an animal/human-based synthetic lure (comprising heptanal, octanal, nonanal, and decanal [Blend F]) and worn socks in a malaria endemic zone in the western part of Kenya. Mosquito Magnet-X (MM-X) and lightless Centre for Disease Control (CDC) light traps were used. Odor-baited traps were compared with traps baited with either solvent alone or solvent + carbon dioxide (controls) for 18 days in a series of randomized incomplete-block designs of days × sites × treatments. The interactive effect of plant and animal/human odor was also tested by combining LO with either Blend F or worn socks. Our results show that irrespective of trap type, traps baited with synthetic plant odors compared favorably to the same traps baited with synthetic animal odors and worn socks in trapping malaria vectors, relative to the controls. Combining LO and worn socks enhanced trap captures of *Anopheles* species while LO + Blend F recorded reduced trap capture. Carbon dioxide enhanced total trap capture of both plant- and animal/human-derived odors. However, significantly higher proportions of male and engorged female *Anopheles gambiae s.l.* were caught when the odor treatments did not include carbon dioxide.

**Conclusion and Significance:**

The results highlight the potential of plant-based odors and specifically linalool oxide, with or without carbon dioxide, for surveillance and mass trapping of malaria vectors.

## Introduction

Malaria continues to be a leading cause of mortality and morbidity in sub-Saharan Africa, with the latest global estimates documenting about 219 million cases in 2010 and an estimated death toll of 1.24 million [1], [2]. Children and pregnant women are the most vulnerable groups, with at least one child dying every minute from malaria in Africa [1], [3]. Though substantial gains have been made in reducing malaria transmission, the death toll from this disease still remains unacceptably high, and as such there is a renewed effort to reduce the disease burden further and move towards malaria eradication [4]. Various control measures have been put in place to help curb this disease. These include chemotherapy, development of malaria vaccines, and reduction of human-vector contact through bed nets and vector-population control. The use of artemisinin-based combination therapy (ACT) is advocated in treatment of clinical cases, but this approach is threatened by recent discovery of emergence of resistant strains of the *Plasmodium* parasite to artemisinin [5], [6]. Efforts have been dedicated to the development of a malaria vaccine, which is viewed as a potent tool to reduce and even eliminate malaria. However, the development of an effective vaccine has been hampered by the complexity of the parasite and its life cycle [7], [8], extensive antigenic variation [9], and a poor understanding of the interaction between *Plasmodium falciparum* and the human immune system [10]. In view of this situation, a multifaceted approach to malaria control is advocated [11], with vector control forming an integral component of it [12]–[14].

Some of the vector control tools that have been used widely with considerable success include long-lasting insecticide-treated nets (LLINs) and indoor residual sprays (IRSs) [15]–[17]. However, the future of vector control based on the use of LLINs or spraying insecticides indoors is uncertain and is threatened by shortage of funds, poor bed-net coverage in some communities, as well as the development of insecticide-resistant mosquitoes [12], [15], [16]. This is exemplified by the resurgence of malaria vectors in sentinel sites in Kenya and elsewhere, despite high ownership and use of LLINs, and by the discovery of outdoor biting fractions of *Anopheles gambiae* Giles [18]–[20]. Outdoor biting among populations of *An. gambiae* is particularly of a serious concern, as they are not susceptible to current indoor control tools and are thus responsible for sustained malaria transmission [13], [21]. This has prompted the need for new and more environmentally robust methods that can supplement the existing vector control methods. New scientific knowledge about the ecology and behavior of mosquitoes and their natural predators and pathogens may lead to the development of new tools that can be incorporated into integrated vector management (IVM) programs. Other approaches that have been developed and are currently being explored for their potential to reduce vector populations include larvicides such as *Bacillus thuringiensis israelensis* (Bti) and insect growth regulators such as methoprene and pyroproxyfen, adulticides such as entomopathogenic fungi and viruses, introduction of genetically engineered and *Wolbachia*-infected mosquitoes, and the sterile insect technique (SIT) [22]–[30]. Despite the availability of these arsenals against the malaria vector, the malaria burden still remains unacceptably high.

Exploiting vector ecology to improve vector surveillance and control has been proposed as a potential new target in the fight against malaria [12], [31]–[33]. Appropriate vector control solutions are highly dependent on the local behavior and ecology of malaria vectors, hydrological and microclimatic conditions, and patterns of disease transmission [34]. Following the successful reduction of tsetse fly populations in parts of Africa [35], the development of an odor-bait technology as a surveillance and control tool for mosquito vectors has been advocated as a new and viable component of IVM [36], [37]. Currently, there are efforts to develop odor-baited traps for control of outdoor biting malaria vectors [38] with the recent development of a mosquito landing box that employs the principle of lure and kill [39]. Up to now, efforts in developing odor-baits for malaria vector management have centered mainly on human/animal-derived odors [36], [40]–[44], which though effective are limited in that they mainly target blood-seeking female mosquitoes [45]. One area that is understudied is the chemical ecology of plant feeding in *Anopheles* mosquitoes, which offers a promising new target for vector control [12], [45]–[47]. Besides the potential to trap mosquitoes of varying physiological status and sexes, plant-odor attraction also offers a unique opportunity to catch malaria vectors outdoors and thus reduce human-vector contact [45]. Furthermore, there have been concerns about the use of carbon dioxide in mosquito traps, since the synthetic forms of CO_2_ supplied via gas cylinders, dry ice, propane combustion, or sugar fermentation are expensive and present logistical challenges for use in remote areas [48]. Given that plants and fruits normally release low amounts of CO_2_ at night as by-products of respiration [49], [50], mosquitoes are not expected to rely heavily on it for host-plant location. Hence, plant-based odors present the potential to minimize or even eliminate the reliance on CO_2_ for trapping, if well formulated.

In an earlier study, we described the performance of an attractive plant-odor blend for *An. gambiae sensu stricto* in laboratory assays [51]. We designed the current study to evaluate the attractiveness of this blend and specific components of it against host-seeking mosquitoes in western Kenya, with emphasis on the outdoor populations of the malaria vectors *An. gambiae s.l.* and *An. funestus* Giles *s.l.* For comparative purposes, we included a newly developed blend of attractants formulated from animal and human odor-based compounds [44] and human foot odor collected on nylon socks (highly attractive for *An. gambiae s.s.* [52]). This allowed us to compare the trapping efficacy of the plant-and animal/human-derived odors in catching malaria mosquitoes of different physiology and age.

## Materials and Methods

### Study site

Preliminary field trials were carried out at *icipe*'s Duduville campus, Nairobi, a low-risk malaria area [53], while field evaluation of optimized blends was conducted at Ahero, located approximately 24 km south-east of Kisumu, in western Kenya (0°10′S, 34°55′E). Malaria is highly endemic in this region and transmission occurs throughout the year. Mean annual *P. falciparum* sporozoite inoculation rates (EIR) of 0.4 -17.0 infective bites per year have been shown by recent studies for this region[54]. The region has an annual mean temperature range of 17–32°C, average annual rainfall of between 1,000–1,800 mm, and average relative humidity of 65% [55].

### Ethical considerations

Consent for homesteads to be used in the study was approved by the Ethical Review Committee at the Kenya Medical Research Institute (Protocol KEMRI/RES/7/3/1) and further from the household heads and the local administration prior to the start of the study.

### Optimization of attractive blends

Six behaviorally-active plant-based compounds reported in our previous study [51] were evaluated individually for trapping wild mosquitoes at three different concentrations to obtain the most attractive concentration. They were prepared in a pentane solvent, starting with the optimal attractive concentration determined from olfactometer studies, followed by consecutive ten-fold higher concentrations (Table S1). Of these six individual components, in a preliminary field trial only (*E*)-linalool oxide (2 ng/µl; mean  =  11.34±0.46, *P*<0.05) and β-ocimene (1 ng/µl: 9.62±0.36, *P*<0.05) caught significant numbers of mosquitoes compared to the control (solvent + carbon dioxide: 2.56±0.62). Based on this trial, three groups of compounds were formulated: (*E*)-linalool oxide only (0.2 ng/µl) (99.5% furanoid form; 0.5% pyranoid form by GC-MS on both methyl silicone and carbowax columns) (hereafter referred to as LO); (*E*)-linalool oxide (0.2 ng/µl) and β-ocimene (0.1 ng/µl) (hereafter referred to as LO + OC); and a blend consisting of all six compounds, i.e. (*E*)-linalool oxide (0.2 ng/µl), β-ocimene (0.1 ng/µl), hexanal (0.2 ng/µl), β-pinene (0.2 ng/µl), limonene (0.2 ng/µl) and (*E*)-β-farnesene (0.1 ng/µl) (hereafter referred to as Blend C) (Table S1). These blends were further evaluated at three different concentrations with ten-fold increase in concentrations (Table S1). Carbon dioxide-baited CDC traps (model 512, John W Hock, Gainesville, FL, powered by 6-V, 10-ampere-hour rechargeable gel-cell battery) without a light bulb, combined with these synthetic chemicals, were deployed in these preliminary studies. Of the three treatments, the second concentrations were the most attractive (i.e. LO [2 ng/µl (*E*)-linalool oxide]; LO + OC [2 ng/µl (*E*)-linalool oxide + 1 ng/µl β-ocimene]; and Blend C [2 ng/µl (*E*)-linalool oxide + 1 ng/µl β-ocimene + 2 ng/µlhexanal +2 ng/µl β-pinene + 2 ng/µl limonene + 1 ng/µl (*E*)-β-farnesene]; Figure S1). These concentrationswere subsequently used in a field evaluation of these plant compounds.

### Field evaluation of optimized blends

Based on the preliminary studies above, only the optimal concentration of the three groups of compounds (LO, LO + OC, and Blend C) were tested in the field alongside controls (solvent only or solvent + CO_2_) and Blend F (developed by Tchouassi *et al.* [44] based on animal and human odors; heptanal  =  2 µg/µl, octanal  =  0.5 µg/µl, nonanal  =  0.1 µg/µl and decanal  =  0.1 µg/µl) and human foot odors. The human foot odors (hereafter referred to as socks) were collected by allowing two volunteers to wear nylon socks for 10–12 h during the day prior to the experiments and replaced every night. The volunteers' feet were cleaned well with non-perfumed soap before wearing the pair of socks and no diet restriction was placed on the volunteers. The synthetic standards of the following compounds were used: hexanal (Aldrich, 98%), β-pinene (Chemika, 99.5%), β-ocimene (Chemika, (*Z*)-β-ocimene  = 27%, (*E*)-β-ocimene  = 67% and allo-ocimene  = 6%), limonene (Sigma), (*E*)-linalool oxide (Aldrich, furanoid form, 99.5% pyranoid form ∼0.5%), (*E*)-β-farnesene (Bedoukian Research, CT, USA), heptanal (Sigma-Aldrich, 98%), octanal (Sigma-Aldrich, 98%), nonanal (Sigma-Aldrich, 98%), and decanal (Sigma-Aldrich, 98%). The odor treatments were evaluated either alone or baited with CO_2_ released as sublimated dry ice from Igloo thermos containers (2 L; John W Hock, Gainesville, FL) with a 13-mm hole in the bottom center (41±2.3 g/h release rate). Two types of traps were used: CDC light trap without the light bulb, with the odors released from 1.5 ml centrifuge tubes (Fisherbrand Scientific, UK) with a pinhole opening; and Mosquito Magnet-X (MM-X) traps (American Biophysics Corporation, RI, USA), with the lure dispensed from a Luna dental roll (Roeko, Langenau, Germany). All field experiments were carried out for 12 h between 18:00 h and 06:00 h local time. In general, odor-baited traps (CDC without light and MM-X) were compared with traps baited with either solvent alone (pentane; for non-CO_2_ baited odors) or solvent + carbon dioxide (for CO_2_-baited odors). The odor treatments were tested for a total of 18 nights for two separate seasons (12 nights in December, 2012 and 6 nights in November 2013) with each night used as a replicate. They were tested in a series of randomized incomplete-block designs of days × sites × treatments at three different sites. Sites were 150–200 m from each other (chosen based on the distribution of potential *An. gambiae s.l.* breeding sites and homesteads), while inter-trap distance was arbitrarily chosen at 20 m apart. Trapping at the three sites was alternated such that every site was sampled every two nights to allow the mosquito population to stabilize. The odor treatments were also rotated within every site to account for positional bias. Carbon dioxide was added nightly by placing 1 kg of dry ice in the containers. To test the significance of CO_2_ in host-plant location by the malaria vectors, the trapping efficacy of CO_2_ only, linalool oxide only, and (*E*)-linalool oxide + carbon dioxide were also evaluated. Traps were removed every morning, the mosquitoes captured were morphologically identified to species or species complex using taxonomic keys [56], [57], and their counts were recorded.

### Interactive effect of plant and human/animal related odors

In another set of experiments, we evaluated the interactive effect of plant and animal/human odors by comparing the trapping efficacy of LO, Blend F, socks, LO + Blend F, and LO + socks either with or without CO_2_ in MM-X traps. The trap captures were compared to worn socks or worn socks + CO_2_ as positive controls for experiments with odor treatments baited with or without CO_2_, respectively. Following a series of randomized incomplete blocking designs, field evaluations were performed at the three sites in a series of randomized incomplete- block designs of days × sites × treatments at three different sites. Sites were 150–200 m from each other (chosen based on the distribution of potential *An. gambiae s.l.* breeding sites and homesteads), while inter-trap distance was arbitrarily chosen at 20 m apart. Trapping at the three sites was alternated such that every site was sampled every two nights to allow the mosquito population to stabilize. The positions of odor treatments at each site were randomly rotated per night to account for any positional bias and each night considered a replicate (total nine replicates). Trapping at the three sites was staggered such that each site was sampled after every two nights to allow the mosquito population to stabilize. Mosquitoes were morphologically identified to species or species complex as described above and their numbers recorded.

### PCR identification of member species of *Anopheles gambiae* complex

Thirty female *An. gambiae s.l.* from each trap treatment were selected and analyzed further to determine the sibling species of the complex. Genomic DNA of each individual *An. gambiae s.l*. mosquito was extracted by homogenizing a leg in 50 µl of sterile double-distilled water in a 1.5 ml Eppendorf tube using a sterile plastic pestle. The homogenates were then boiled for 45 min ona water bath, allowed to cool and kept at −20°C until required. The PCR method of Scott et al. [58] was used for the identification of the sibling species of the *An. gambiae* complex. Amplicons were visualized in ethidium bromide-stained 2% agarose gels.

### Physiological state of field collected mosquitoes

Mosquitoes were scored as unfed, blood-fed or half-gravid/gravid, based on appearance of their abdominal condition as illustrated in the WHO Manual [59]. Based on the risk of disease transmission, any *An. gambiae s.l.* or *An. funestus s.l.* mosquito with prior encounter with a blood host (either blood-fed or half-gravid/gravid) were categorized as engorged and otherwise as unfed. The number of male *An. gambiae s.l.* and *An. funestus s.l.* was also recorded for each treatment. Other *Anopheles* species (*An. coustani* Laveran and *An. pharoensis* Theobald) were neither classified by sex nor abdominal status.

### Statistical analyses

The total trap captures of MM-X and CDC traps was compared using chi-square goodness-of-fit test. The numbers of mosquitoes per treatment were analyzed using a generalized linear model (GLM) with negative-binomial error structure and log link in R 2.15.1 software [60]. This model assumes a chi square distribution suitable for count data [61]. With the solvent-only or the solvent + CO_2_-baited CDC or MM-X trap (control) serving as the reference category, we calculated the incidence-rate ratios (IRR), a measure of the likelihood that mosquito species chose treatments other than the control, as well as their *P-*values and confidence intervals, from the model. A similar model was used to compare the performance of plant- and animal/human-based odors separately and when combined with worn socks used as control. The proportions of male and engorged female *An. gambiae s.l.* and *An. funestus s.l.* caught by the different odor treatments relative to worn socks were compared using chi-square test of proportions.

## Results

### Field evaluation of optimized blends

Overall, MM-X traps caught 2.8 times more mosquitoes thanCDC traps (CI  = 2.75–2.92; *P*<0.001) (Table 1). There was a significant increase in trap capture when traps were baited with plant-derived odors, animal-derived odors or worn socks compared to the control solvent or solvent + CO_2_. In the absence of carbon dioxide, MM-X traps baited either with plant-derived odors, Blend F or worn socks had significantly higher captures of both *An. gambiae s.l.* and *An. funestus s.l.* than the control (solvent) (χ^2^ = 171.85, df  = 96, *P*<0.001 and χ^2^ = 47.304df  = 96, *P*<0.001, respectively) (Table 2). Similarly, MM-X traps baited with any of these odor treatments in the presence of CO_2_ had significantly higher captures of both *An. gambiae s.l.* and *An. funestus s.l.* compared to traps baited with solvent + CO_2_ (χ^2^ = 348.5, df  = 96, *P*<0.001 and χ^2^ = 107.12, df  = 96, *P*<0.001, respectively) (Table 2).

**Table 1:** Total number of each mosquito species/genusof both sexes caught by each type of trap.

Species	CDC	MM-X	χ2	*P*-value
*An. gambiae s.l.*	229	1655	2155.65	<0.001
*An. funestus s.l.*	230	867	737.46	<0.001
Other *Anopheles* spp.	11	1010	1951.03	<0.001
*Culex* spp.	1205	7847	9297.50	<0.001
*Mansonia* spp.	4201	5080	166.12	<0.001
Other mosquito spp.	15	238	389.60	<0.001

**Table 2:** Total anopheline trap captures with plant and animal odor compounds and worn socks in the presence or absence of carbon dioxide using MM-X traps.

Species	Treatment	N	Without CO_2_			With CO_2_		
			n	IRR (95% CI)	*P-*value	n	IRR (95% CI)	*P-*value
*An. gambiae s.l.*	Control	18	23	1.0		47	1.0	
	LO	18	188	8.9 (5.85–14.48)	<0.001	275	10 (7.05–15.59)	<0.001
	LO+OC	18	88	3.8 (2.40–6.32)	<0.001	228	8.5 (5.80–12.93)	<0.001
	Blend C	18	64	3.0 (1.90–5.11)	<0.001	185	6.9 (4.66–10.49)	<0.001
	Blend F	18	53	3.6 (2.40–5.94)	<0.001	228	8.4 (5.78–12.87)	<0.001
	Socks	18	76	3.7 (2.41–6.02)	<0.001	248	9.7 (6.44–14.97)	<0.001
*An. funestus s.l.*	Control	18	12	1.0		33	1.0	
	LO	18	61	5.1 (2.84–9.91)	<0.001	115	3.5 (2.40–5.21)	<0.001
	LO+OC	18	33	2.9 (1.56–5.86)	<0.01	127	3.7 (2.57–5.56)	<0.001
	Blend C	18	51	4.3 (2.35–8.36)	<0.001	127	3.8 (2.66–5.73)	<0.001
	Blend F	18	59	4.9 (2.74–9.60)	<0.001	86	2.7 (2.01–5.35)	<0.001
	Socks	18	35	3.1 (1.54–5.79)	<0.001	98	2.9 (2.04–4.70)	<0.001

IRR  =  incidence rate ratio, CI  =  confidence interval, N  =  number of replicates, n  =  total number of mosquitoes caught, LO  =  (*E*)-linalool oxide and OC  =  β-ocimene. NB: Control (solvent or solvent + CO2) was used as reference.

The efficacy of the plant- and animal-derived odors as well as worn socks in trapping *An. gambiae s.l.* and *An. funestus s.l.* was further demonstrated in the lightless CDC traps (Table 3). While only traps baited with Blend C and Blend F registered significant increase in trap captures of *An. gambiae s.l.* compared to the control solvent (χ^2^ = 29.911, df  = 107, *P*<0.001), none of these baited traps performed better than the control in trapping *An. funestus s.l.* in the absence of CO_2_. On the other hand, all the odor treatments had significantly higher captures of both *An. gambiae s.l.* and *An. funestus s.l.* in the presence of CO_2_ relative to the control (χ^2^ = 64.798, df  = 107, *P*<0.001 and χ^2^ = 32.472, df  = 107, *P*<0.001, respectively) (Table 3).

**Table 3:** Total anopheline trap captures with plant and animal odor compounds and worn socks in the presence or absence of carbon dioxide using CDC traps.

Species	Treatment	N	Without CO_2_			With CO_2_		
			n	IRR (95% CI)	*P-*value	n	IRR (95% CI)	*P-*value
*An. gambiae s.l.*	Control	18	11	1.0		17	1.0	
	LO	18	19	1.4 (0.68–3.04)	0.36	80	3.9 (2.34–7.05)	<0.001
	LO+OC	18	13	1.0 (0.44–2.25)	1.00	85	4.9 (2.97–8.76)	<0.001
	Blend C	18	38	3.2 (1.71–6.33)	<0.001	55	2.9 (1.70–5.34)	<0.001
	Blend F	18	30	2.5 (1.31–5.08)	<0.01	80	5.0 (3.01–8.87)	<0.001
	Socks	18	9	0.9 (0.40–2.09)	0.83	77	4.8 (2.89–8.55)	<0.001
*An. funestus s.l.*	Control	18	9	1.0		10	1.0	
	LO	18	14	1.1 (0.49–2.41)	0.84	42	2.7 (1.46–5.28)	<0.01
	LO+OC	18	13	0.9 (0.40–2.09)	0.84	61	4.4 (2.48–8.37)	<0.001
	Blend C	18	13	1.0 (0.44–2.25)	1.00	31	2.0 (1.05–4.02)	<0.05
	Blend F	18	15	1.3 (0.59–2.72)	0.57	28	2.2 (1.14–4.30)	<0.05
	Socks	18	9	0.8 (0.35–1.93)	0.67	26	2.3 (1.23–4.57)	<0.05

IRR  =  incidence rate ratio, CI  =  confidence interval, N  =  number of replicates, n  =  total number of mosquitoes caught, LO  =  (*E*)-linalool oxide and OC  =  β-ocimene. NB: Control (solvent or solvent + CO_2_) was used as a reference.

**Figure 1:**
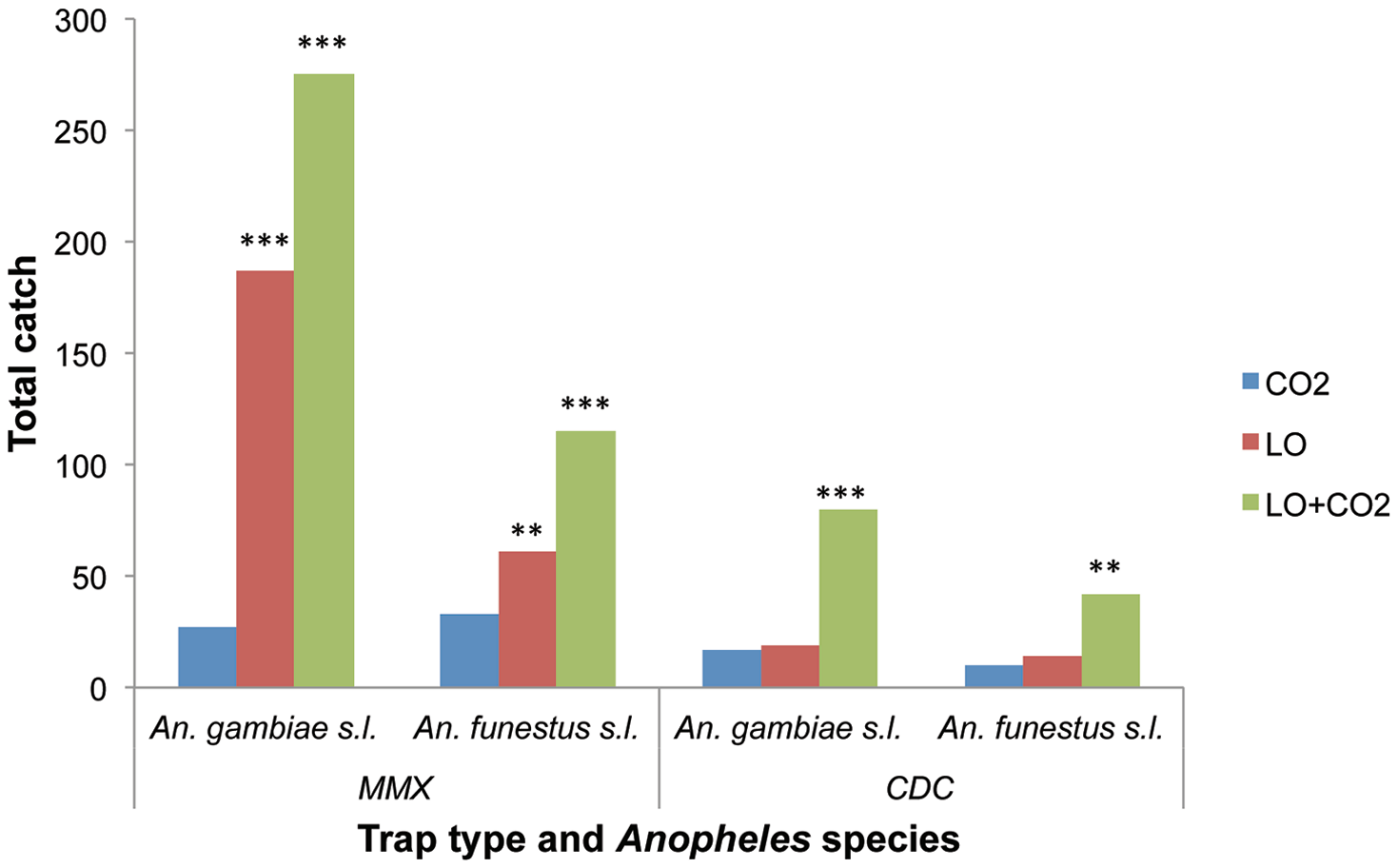
Trapping efficacies of carbon dioxide and linalool oxide for *An. gambiae s.l.* and *An. funestus s.l.* . Number of replicates  = 18; bars capped with asterisks are significantly different from their respective controls as determined by general linear model with negative-binomial error structure and log link in R 2.15.1 software; **  = *P*<0.01, ***  = *P*<0.001.

Further analysis revealed that in the absence of carbon dioxide, LO was superior than any of the other odor treatments in trapping the two malaria vectors and was as good as any of them when used together with CO_2_ (Tables 2 and 3). When LO was compared with CO_2_, it trapped 7- and 1.8-fold more *An. gambiae s.l.* and *An. funestus s.l.*, respectively, than CO_2_ when dispensed using MM-X traps but not CDC traps (Figure 1). Combining LO and CO_2_ enhanced trap captures for both MM-X and CDC traps (Figure 1). The enhanced effect of CO_2_ with plant and animal/human odors was further confirmed for LO+OC, Blend C, Blend F and socks, which registered significantly increased captures of the two mosquito species compared to non-carbon dioxide baited traps (Tables 2 and 3).

### Interactive effect of plant and human/animal related odors

Combining LO with worn socks significantly increased the trap captures of *An. gambiae s.l.* both in the absence and presence of CO_2_ (χ^2^ = 2.12, df  = 46, *P*<0.05 and χ^2^ = 4.35, df  = 46, *P*<0.001, respectively) but this did not affect the number of *An. funestus s.l.* trapped (Table 4). On the other hand, combining LO with Blend F significantly reduced trap captures of both *An. gambiae s.l.* and *An. funestus s.l.*; with significant reduction in trap captures of *An. funestus s.l*. recorded in non-CO_2_-baited traps (χ^2^ = 2.38, df  = 46, *P*<0.05) (Table 4).

**Table 4:** Interactive effect of plant and animal/human lures on anopheline trap captures using MM-X traps.

Species	Treatment	N	Without CO_2_			With CO_2_		
			n	IRR (95% CI)	*P-*value	n	IRR (95% CI)	*P-*value
*An. gambiae s.l.*	Socks	9	48	1.0		166	1.0	
	LO	9	90	1.7 (1.21–2.49)	<0.01	237	1.4 (1.15–1.71)	<0.001
	Blend F	9	69	1.4 (0.95–2.02)	0.09	181	1.1 (0.88–1.34)	0.42
	LO+Blend F	9	62	1.3 (0.90–1.94)	0.15	152	0.9 (0.73–1.14)	0.43
	LO+Socks	9	79	1.4 (1.00–2.11)	<0.05	256	1.5 (1.27–1.88)	<0.001
*An. funestus s.l.*	Socks	9	23	1.0		34	1.0	
	LO	9	26	1 (0.56–1.79)	1.00	32	0.9 (0.54–1.44)	0.62
	Blend F	9	36	1.5 (0.91–2.61)	0.18	78	2.3(1.55–3.47)	<0.001
	LO+Blend F	9	11	0.4 (0.17–0.82)	<0.05	36	1.1 (0.66–1.70)	0.81
	LO+Socks	9	28	1.2 (0.67–2.06)	0.57	33	1 (0.60–1.57)	0.90

IRR  =  incidence rate ratio, CI  =  confidence interval, N  =  number of replicates, n  =  total number of mosquitoes caught, LO  =  (*E*)-linalool oxide and OC  =  β-ocimene. Worn socks were used as reference.

### PCR identification of member species of *Anopheles gambiae* complex

Of the 501 *An. gambiae s.l.* processed for molecular species identification, 99% (n = 496) were identified as *An. arabiensis* with only 1% (n = 5) as *An. gambiae s.s.* The few *An. gambiae s.s.* recorded were all found in the MM-X trap baited with Blend F+CO_2_.

### Physiological status of field collected mosquitoes

Irrespective of odor treatment, unfed females constituted the highest percentage of the total number of mosquitoes caught by the two trap types. Of the *Anopheles* species captured, CDC traps captured16 male and 442 female *An. gambiae s.l.* (89% unfed, 8% blood-fed and 3%half-gravid/gravid); and 14 male and 265 female *An. funestus s.l.* (95% unfed, and 5% blood-fed), while MM-X traps captured 77 male and 1575 female *An. gambiae s.l.* (84% unfed, 11 blood-fed % and 5% gravid); and 52 male and 822 female *An. funestus s.l.* (91% unfed, 2% blood-fed and 7% gravid).

**Figure 2:**
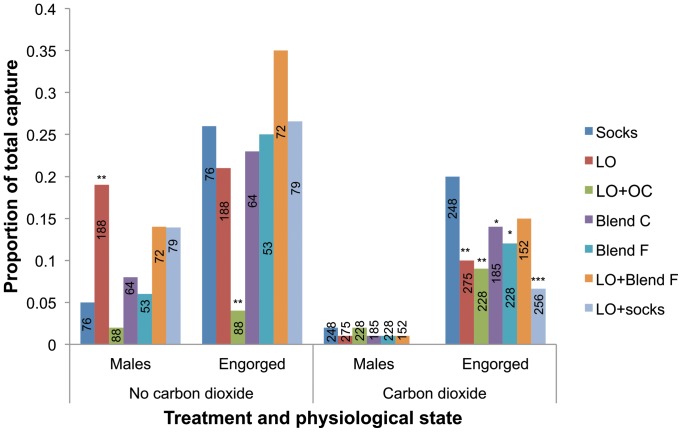
Proportions of male and engorged female *An. gambiae s.l.* caught by different odor treatments. The bars show the proportions of male and engorged (blood-fed + semi-gravid/gravid) female *An. gambiae s.l.*; numbers embedded in the bars are the total of mosquitoes caught by each of the odor treatment; LO  =  (*E*)-linalool oxide; OC  =  β-ocimene; the different treatments were compared to socks (control); bars capped with asterisks are significantly different from their respective controls as determined by chi square test of proportions in R 2.15.1 software *  = *P*<0.05, **  = *P*<0.01, ***  = *P*<0.001.

To understand the diversity of the various physiological states of mosquitoes caught by the plant- and animal/human-based odors, we analyzed the proportions of male and engorged (blood-fed + half-gravid/gravid) female *An. gambiae s.l.* caught by the different odor treatments. Overall, the odor treatments caught a higher proportion of male and engorged female *An. gambiae s.l.* in the absence of CO_2_ than when they were combined with carbon dioxide (Figure 2). In the absence of CO_2_, LO-baited traps captured a significantly higher proportion of male *An. gambiae s.l.* than the traps baited with worn socks (χ^2^ = 6.66, df  = 1, *P*<0.01) while the trap baited with LO+OC had a significantly lower proportion of engorged females than the traps baited with worn socks (χ^2^ = 9.85, df  = 1, *P*<0.01) (Figure 2). On the other hand, traps baited with worn socks caught a significantly higher proportion of engorged female *An. gambiae s.l.* than those baited with LO, LO+OC, Blend C and Blend F (Figure 2). Combining LO with either Blend F or worn socks did not significantly affect the performance of Blend F, but in the presence of CO_2_ it significantly reduced the proportion of engorged female *An. gambiae s.l.* trapped by the worn socks compared to when the socks were used with CO_2_ alone (χ^2^ = 18.82, df  = 1, *P*<0.001).

**Figure 3:**
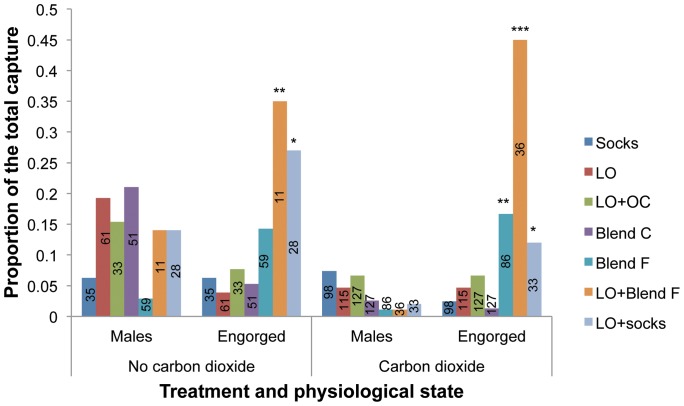
Proportions of male and engorged female *An. funestus s.l.* caught by different odor treatments. The bars show the proportions of male and engorged (blood-fed + semi-gravid/gravid) female *An. funestus s.l.*; numbers embedded in the bars are the total of mosquitoes caught by each of the odor treatment; LO  =  (*E*)-linalool oxide; OC  =  -β-ocimene; the different treatments were compared to socks (control); bars capped with asterisks are significantly different from their respective controls as determined by chi square test of proportions using R 2.15.1 software *  = *P*<0.05, **  = *P*<0.01, ***  = *P*<0.001.

In terms of *An. funestuss.l.* captured, only traps baited with Blend F, LO + Blend F, and LO + socks caught a significant number of engorged females compared to socks both in the presence and absence of CO_2_ (Figure 3). Overall, a reduced number of males and engorged female *An. funestus s.l.* were captured in traps when the odors were combined with CO_2_.

## Discussion

Our results show that the two trap types differed in the number of mosquito captures. The MM-X traps, either unbaited or baited with plant-based or animal/human odors, captured a higher number of mosquitoes than the corresponding CDC traps in terms of total mosquito captures as well as *Anopheles* captures. It is likely that the different dispensers used with different release rates of odors could have influenced captures in both traps. As such, these results should be interpreted with caution. However, the difference in designs for both traps supposes that the release rates are likely to be different even for the same type of dispensers. These results corroborate previous findings [62]–[64], which have shown consistently that traps operating under the counter-flow principle are generally more efficient compared to other trap types. It is not surprising therefore, that higher captures were recorded in the MM-X trap which operates using the counter-flow concept with two fans, the weaker one dispersing the odor and the stronger one sucking the mosquitoes into the trap as they are flying up the odor plume [65].

Furthermore, we found that like animal/human-derived odors, plant odors also performed well in trapping malaria vectors, compared to controls. Aside from the field trapping with ripened fruits and flowers [66], [67], this is the first field evidence of malaria vector attraction to plant-derived odors. Particularly outstanding was the performance of LO, which in the absence of CO_2_, had the highest *Anopheles* capture as well as the highest proportion of males. These findings confirm the significance of olfaction in the mosquito-host plant interactions and therefore underpin the potential for deployment of plant-derived odors in IVM. There has been widespread concern about the prospects of malaria eradication in the recent past, following the emergence of a cryptic subgroup of outdoor-biting *An. gambiae*, which is implicated in the sustained transmission of malaria even in communities where LLINs and IRS are regularly implemented [4], [20]. Given that sugar feeding in mosquitoes predominantly takes place outdoors and that mosquitoes use olfactory cues to locate sugar sources [47], [51], these findings are significant as they highlight the potential of deploying these plant odors in controlling sugar-seeking segments of a malaria vector population. Sugar feeding takes place at all states of an adult mosquito [68], hence a wide spectrum of the malaria vector population, including age, sex, and behavioral state, can be targeted.

Furthermore, progress has been made in the development of attractive toxic sugar-baits (ATSB) [69], [70], but these baits are still limited by the fact that they employ ripened fruits and flowering plants, and therefore their application is still restricted to available plant products [69], [70]. Thus, these findings present opportunities for deployment of attractive synthetic plant-derived odors such as linalool oxide to enhance the efficacy of ATSB. Unlike the aldehydes that constituted the animal-derived bait in the present study and can oxidize easily in air, linalool oxide is relatively stable [71]. Therefore, it can be employed in long-term field trappings in remote malaria-endemic villages in Africa where access to dry ice or CO_2_ generators that consume large amounts of sugar can be a problem.

This study also highlights the potential of utilizing a combination of plant-based and animal/human odors in surveillance and control of malaria vectors. While it appeared that there was an inhibitory effect on trap captures when LO was combined with Blend F, an enhanced effect was recorded in trapping *An. gambiae s.l.* when LO was combined with worn socks. The exclusive aldehyde components in Blend F combined with LO seemed to exhibit an antagonistic effect on each other as compared to the socks, which releases a diverse range of chemicals. This suggests that the interactive effects of plant and animal odors might be limited to a certain group or individual compounds. The interactive effect of these lures on mosquito captures has not been evaluated; yet, this approach may open up new ways for maximizing trap lures in order to minimize the use and over-dependence on CO_2_ in surveillance traps.

We also found that irrespective of the trap type and the odor bait, inclusion of CO_2_ significantly increased mosquito trap capture. Carbon dioxide and host odors both play important roles in long-range host selection and orientation, with specific host odors playing a role in close-range host recognition and acceptance [52], [72]. The receptors for CO_2_ are located on the maxillary palps of mosquitoes and have been shown to be sensitive to even as slight a change in atmospheric carbon dioxide concentration as 0.01% [48], [73], [74]. Mosquitoes aregenerally thought to respond to small changes in CO_2_ concentration by flying upwind [75], [76] and to use optomotor anemotaxis to orient to the source of the host odor [77]. These behavioral responses vary with plume structure and odor [78]. Plants normally release small amounts of CO_2_, constituting between 0.01 and 0.1% of atmospheric CO_2_ at night [49]. The combined effect found between CO_2_ and plant odors therefore suggests that possibly Afrotropical malaria vectors also utilize CO_2_ to locate their potential host plants. Alternatively, given the observed paucity of males in the collections and the large amounts of CO_2_ probably being released from the containers, it is likely that it also was serving as a blood-host kairomone. Taken together, these observations and results suggest the possibility that mosquitoes were responding to both plant and animal cues, thus explaining the partial additive effects of their combinations. This interpretation fits with the conclusion that, unlike most zoophilic mosquito species, the Afrotropical malaria vectors are known to rely more on the specific host odors rather than CO_2_ to locate a suitable host [79].

Interestingly, this study also indicates a reduction in the proportions of male and engorged female *An. gambiae s.l.* and *An. funestus s.l.* when the tested odors were combined with CO_2_ as compared to when used alone. While the reduction in proportion of males captured in CO_2_-baited traps is not surprising given that males feed entirely on plant nectar, the reduction in proportions of engorged females was not expected. A possible explanation for this is that carbon dioxide reception in engorged female *Anopheles* triggers an avoidance behavior as opposed to its attractive role in unfed females. This study also shows that in the absence of CO_2_, (*E*)-linalool oxide caught a significantly higher proportion of males than worn socks and compares well with it in trapping engorged females. It also increases the proportion of males caught by socks and Blend F as well as the proportion of engorged females caught by Blend F when combined. This confirms the hypothesis that plant-odor-based attractants have the potential to attract mosquitoes of more divergent physiology and sex [45]. Thus, adoption of a plant-odor-bait technology presents a potent tool for surveillance of malaria vectors of varying physiological states. However, it is important to note that the total number of males was low in both plant- and animal/human-derived odor-baited traps. This result may be explained by the locations of the traps, which were next to homesteads. Possibly, if such traps were to be placed next to breeding sites, they might capture more males, given that mating in *An. gambiae s.l.* and *An. funestus s.l.* occurs a few days after emergence within the vicinity of breeding sites or in swarms formed around specific environmental swarm markers [80]–[82].

While our lures had been optimized for *An. gambiae s.s*. in the laboratory, the field study was dominated by *An. arabiensis* in collections of *An. gambiae s.l.* and *An. funestus s.l.*, both of which are important malaria vectors in western Kenya. There has been a proportionate increase in *An. arabiensis* compared to its closely related sibling species *An. gambiaes.s*. in western Kenya, presumably as a result of widespread bed net use [83]. The exophilic and anthropo-zoophilic tendencies of *An. arabiensis* imply it is less susceptible to bed nets. Our current study shows dominance of trap captures by *An. arabiensis*, which is in line with similar findings by recent studies [40], [43]. The significant response to the lures by *An. funestus s.l.*, which together with *An. arabiensis* constitute important malaria vectors, suggests that our lures used here can provide a tool for the sampling of these species. Given that sugar resources are readily exploited by most mosquito species, the significantly increased captures recorded for the plant-based lure emphasizes its role as an attractant for a wide range of mosquito species as evidenced by the diversity of mosquito species collected in this study.

## Conclusion

This field study has confirmed the effectiveness of plant-derived synthetic odors in trapping malaria vectors and other mosquito species. The study also highlights the potential of combining plant and human odors in trapping malaria vectors, as well as the role played by CO_2_ in trapping *An. gambiae s.l.* and *An. funestus s.l.* of varying physiology and sex. Traps baited with plant-derived chemicals significantly increased capture of female mosquitoes when compared to either CDC or MM-X traps baited with either solvent or solvent + CO_2_. Thus, the use of plant-produced kairomones has significant promise for the surveillance and integrated control of malaria vector populations in Africa.

## Supporting Information

Figure S1 **Preliminary trap captures of all species of mosquitoes using different concentrations of plant-derived synthetic blends.** LO  =  (*E*)-linalool oxide, OC  =  β-ocimene, bars capped with asterisks are significantly different from their respective controls as detected by general linear model with negative-binomial error structure and log link in R 2.15.1 software; *  =  *P*<0.05, **  =  *P*<0.01.
(PNG)

Table S1 **Composition and concentrations of blends tested in field assays.**
(DOCX)
