# Supplementary figures and images for: Development and Assessment of Plant-Based Synthetic Odor Baits for Surveillance and Control of Malaria Vectors
Source: PLoS One. 2014 Feb 24;9(2):e89818. doi: 10.1371/journal.pone.0089818 (PMC3933673; doi:10.1371/journal.pone.0089818)

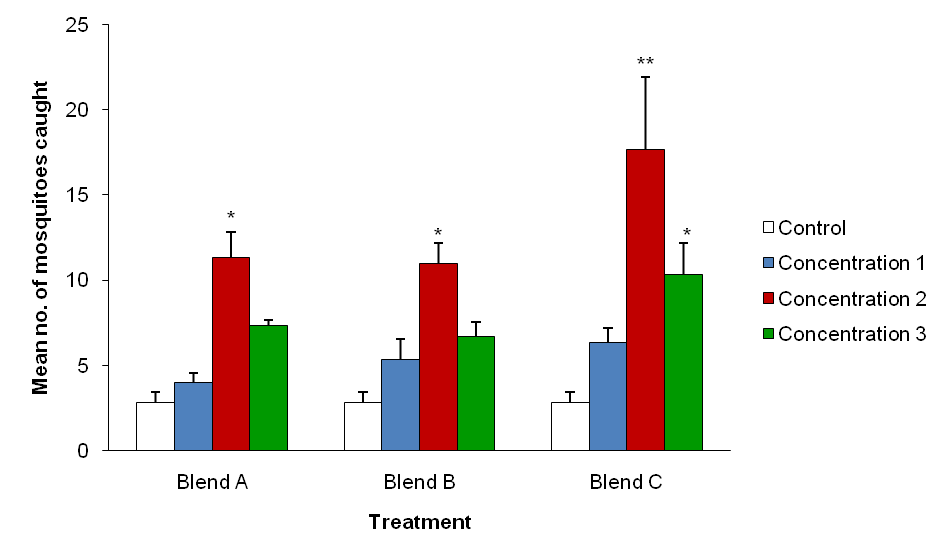

Supplement: Figure S1 — Preliminary trap captures of all species of mosquitoes using different concentrations of plant-derived synthetic blends. LO = (E)-linalool oxide, OC = β-ocimene, bars capped with asterisks are significantly different from their respective controls as detected by general linear model with negative-binomial error structure and log link in R 2.15.1 software; * = P<0.05, ** = P<0.01. (PNG) [file pone.0089818.s001.png]
