# Supplementary material for: Development and Assessment of Plant-Based Synthetic Odor Baits for Surveillance and Control of Malaria Vectors
Source: PLoS One. 2014 Feb 24;9(2):e89818. doi: 10.1371/journal.pone.0089818 (PMC3933673; doi:10.1371/journal.pone.0089818)
Supplement: Table S1 — Composition and concentrations of blends tested in field assays. (DOCX) [file pone.0089818.s002.docx]

**Table S1** Composition and concentrations of blends tested in field assays.

| **Compound** | **Concentration**  **(ng/µl)** | **Blend A** | | | **Blend B** | | | **Blend C** | | |
| --- | --- | --- | --- | --- | --- | --- | --- | --- | --- | --- |
|  |  | 1 | 2 | 3 | 1 | 2 | 3 | 1 | 2 | 3 |
| (*E*)-Linalool oxide | 0.2 | √ | - | - | √ | - | - | √ | - | - |
|  | 2 | - | √ | - | - | √ | - | - | √ | - |
|  | 20 | - | - | √ | - | - | √ | - | - | √ |
| Hexanal | 0.2 | - | - | - | - | - | - | √ | - | - |
|  | 2 | - | - | - | - | - | - | - | √ | - |
|  | 20 | - | - | - | - | - | - | - | - | √ |
| β-Pinene | 0.2 | - | - | - | - | - | - | √ | - | - |
|  | 2 | - | - | - | - | - | - | - | √ | - |
|  | 20 | - | - | - | - | - | - | - | - | √ |
| Limonene | 0.2 | - | - | - | - | - | - | √ | - | - |
|  | 2 | - | - | - | - | - | - | - | √ | - |
|  | 20 | - | - | - | - | - | - | - | - | √ |
| (*E*)-β-Ocimene | 0.1 | - | - | - | √ | - | - | √ | - | - |
|  | 1 | - | - | - | - | √ | - | - | √ | - |
|  | 10 | - | - | - | - | - | √ | - | - | √ |
| (*E*)-β-Farnesene | 0.1 | - | - | - | - | - | - | √ | - | - |
|  | 1 | - | - | - | - | - | - | - | √ | - |
|  | 10 | - | - | - | - | - | - | - | - | √ |

Check marks (√) indicate the compounds and concentrations present in the blends.
